# Analytical considerations when using the Scottish and English Cancer Registries for applied research: the case of colorectal cancer.

**DOI:** 10.23889/ijpds.v7i3.2049

**Published:** 2022-08-25

**Authors:** Elizabeth Lemmon, Rebecca Birch, Peter Hall, Eva Morris, Amy Downing

**Affiliations:** 1 University of Edinburgh, Edinburgh Health Economics; 2 University of Leeds; 3 University of Edinburgh; 4 University of Oxford

In this paper, we carry out a retrospective analysis of the Scottish and English cancer registries for the case of colorectal cancer. We aim to assess the comparability of variable coding between the datasets and create derived variables and open access code for use by researchers working with linked Scottish and English cancer registry data.

We bring together the national data dictionaries for the Scottish and English cancer registries and define a list of targeted variables. We establish the consistency of variable coding across the two registries, identifying fully complete, near complete, partial, composite and impossible matched variables. In the case of near complete, partial and composite matches, we create derived variables between the two registries. We liaise with NHS staff working within the retrospective registry teams to validate the derivations. Finally, we produce corresponding documentation for the dissemination and preservation of final data items.

We considered 63 variables for analysis. Preliminary results show that there is a high degree of similarity between the Scottish and English cancer registries. In particular, 82% of variables were fully, nearly or partially matched, with the remaining 12% and 6% composite matches and impossible matches respectively. The session with the respective cancer registry teams will take place in May 2022. At which point we will present our existing results and seek their input into the final derived data items. Following this, we will publish documentation detailing the derived variables and associated code, to be made available to other researchers working with the Scottish and English cancer registries.

This study provides a useful starting point for any researchers seeking to use the linked Scottish and English cancer registry data for applied research. Whilst our analysis has focused on colorectal cancer, the majority of variables are applicable for any cancer. Future research should extend this work to include staging variables for other cancers.

